# Efficiency of generic and proprietary inhibitors in mitigating Corrosion of Carbon Steel in Chloride-Sulfate Environments

**DOI:** 10.1038/s41598-018-29413-7

**Published:** 2018-07-30

**Authors:** Khaled A. Alawi Al-Sodani, Mohammed Maslehuddin, Omar S. Baghabra Al-Amoudi, Tawfik A. Saleh, Mohammed Shameem

**Affiliations:** 1 Department of Civil and Environmental Engineering, King Fahd University of Petroleum & Minerals, Dhahran, 31261 Saudi Arabia; 2 Center for Engineering Research, Research Institute, King Fahd University of Petroleum & Minerals, Dhahran, 31261 Saudi Arabia; 3 Department of Chemistry, King Fahd University of Petroleum & Minerals, Dhahran, 31261 Saudi Arabia; 4 Present Address: The Department of Civil Engineering, University of Hafr Al Batin, Hafr Al Batin, 31991 Saudi Arabia

## Abstract

The efficiency of generic and proprietary corrosion inhibitors (based on nitrite, amine carboxylate or amino alcohol) in corrosion mitigation of carbon steel, which is exposed to concrete solutions with different amounts of chloride as well as sulfate, was studied. The corrosion protection provided by the selected corrosion inhibitors was investigated by performing a potentiodynamic polarization study. In addition, the surface morphological properties of carbon steel samples exposed to the electrolyte mixed with or without inhibitors was also evaluated by scanning electron microscopy. The potentiodynamic polarization measurements showed that the evaluated inhibitors decreased the corrosion current density by 1.6 to 6.7 times depending on the type of inhibitor and the level of sulfate concentration in the electrolyte. The performance of inhibitors based on nitrite was better than that of inhibitors based on amine carboxylate or amino alcohol. The possible mechanisms of the inhibition in the chloride plus sulfate environments are also elucidated.

## Introduction

Reinforced concrete was once presumed to be a maintenance-free construction material. However, concrete durability problems have been noted in certain situations, resulting in significant reduction in the expected lifetime of structures. The deterioration is mainly attributed to improper construction practices, inappropriate materials selection, harsh service conditions, inferior design, or a combination thereof^1^. Substantial resources have to be, therefore, diverted towards the rehabilitation and repair of the deteriorated reinforced concrete structures^1^. Reinforced concrete structures are sometimes affected by many processes leading to the loss of serviceability, or in extreme cases, to structural collapse. Reinforcement corrosion, sulfate attack, alkali-aggregate reaction, and freeze-thaw damage are some of the commonly occurring concrete durability problems^1–4^. However, corrosion of reinforcing steel is considered to be the major cause of concrete deterioration^1,5–7^. Apart from carbonation of concrete, which is a rather slow process and constitutes a real problem in industrialized regions and old structures^8^, the diffusion of chloride ions to the steel is the principal cause of reinforcement corrosion^9^. Although reinforcement corrosion can be ascribed to chloride ions, there is a concern with regard to the role of sulfate ions on the pheonomenon^8,10–13^. Sulfate and chloride salts are present in the offshore and marine environment and in certain soils in the coastal and inland regions^14^.

One of the techniques used to minimize reinforcement corrosion is to add a chemical inhibitor to concrete. A number of papers^15–21^ were reported on the utilization of inhibitors in samples of concrete. Most of these studies have concentrated on the effectiveness of the inhibitors in different environments such as concrete contaminated with chloride. The results of earlier studies indicated that benzotriazole is an active inhibitor to avert the chloride-induced corrosion of steel inside a simulated concrete pore solution (SCPS). Sodium nitrite (NaNO_2_) and sodium phosphate (Na_3_PO_4_) were mainly not effective in high chloride environments^22^.

In an earlier study, the corrosion rate of reinforcing steel was measured in solutions simulating electrolytic chloride environments in the presence of NaNO_2_^23^. It was reported that the presence of NaNO_2_ significantly decreased the corrosion rate at low chloride concentrations, although its efficiency decreased as the pH was decreased^23^. A polarization investigation was performed to evaluate the corrosion of mild steel in SCPS prepared with varying types of water^24^. It was reported that the corrosion resistance increased in the following order: Rainwater > Well water > Seawater. The effect of benzotriazole and four other benzotriazole derivatives on the corrosion resistance of the steel in SCPS was evaluated^25^. It was reported that the pitting potential decreased due to the selected protection systems and the selected inhibitors that provided a good level of protection to the steel in SCPS^25^.

A literature review showed that the efficiency of chemical inhibitors has been mainly evaluated for chloride-contaminated concrete^26^. However, their efficiency in environments contaminated with both sulfate and chloride salts and under high exposure temperature has not been adequately evaluated^26^. Accordingly, the reported work was performed to evaluate the efficacy of selected inhibitors, i.e. generic and proprietary, in the presence of sulfate and chloride ions at elevated temperature. The mechanisms of inhibition have also been proposed.

## Experimental

### Corrosion inhibitors

The corrosion inhibitors were selected based on the functional group (nitrite, amine carboxylate and amino alcohol) to assess their efficiency in chloride and sulfate environments. Five (generic and proprietary) corrosion inhibitors were evaluated. A complete presentation of the investigated inhibitors is listed in Table 1, which highlights the designation of the inhibitors properties such as dosage, colour, density and pH^27,28^. In this study, the inhibitors performance was evaluated under the simultaneous presence of sulfate and chloride concentrations at elevated temperature.

**Table 1:** Description of the investigated corrosion inhibitors.

Designation of the inhibitor	Description of the inhibitor	Dosage (L/m^3^ of concrete)	Colour	Density (kg/L)	pH
Inhibitor I	Proprietary liquid concrete mixture based on calcium nitrite	15	Pale straw	1.25–1.3	9.5–11.5
Inhibitor II	Generic corrosion inhibitor based on calcium nitrite	15	Dark yellow	1.28	10.5
Inhibitor III	Proprietary liquid concrete mixture based on amine carboxylate	0.6	Dark brown	1.17–1.23	11–12
Inhibitor IV	Proprietary liquid concrete admixture based on modified amino alcohol	15	Green	1.06	10 ± 1
Inhibitor V	Proprietary liquid concrete admixture based on calcium nitrite	15	Pale yellow	1.22	7 ± 1

### Preparation of Carbon Steel Specimens

**Figure 1:**
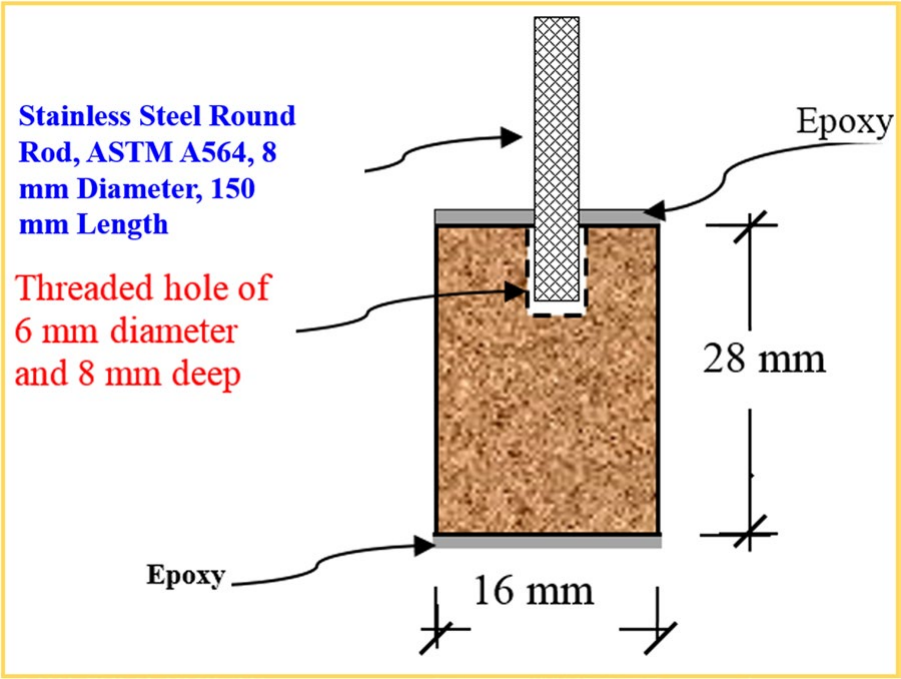
Schematic representation of carbon steel specimens.

Carbon steel conforming to ASTM A706 M was used in the preparation of specimens for the reported electrochemical studies. These type of low-alloy steel deformed bars (Grade 420) are specified for applications where extensive welding of reinforcement or controlled ductility for reinforced concrete structures is required. The specimens were prepared from carbon steel bars obtained from a local steel plant (Saudi Iron and Steel Company). The chemical composition of the carbon steel is shown in Table 2. Test specimens of 16 mm in diameter and 28 mm high were prepared for the electrochemical and morphological studies. The carbon steel samples were cleaned with sandpapers followed by cleaning by acetone. Epoxy coating was used to coat the ends of the specimens to obtain an exposed surface area of 14.6 cm^2^. Figure 1 is a scheme representing the steel sample. A 6 mm diameter hole was drilled in the top surface of the carbon steel sample in order to fit a thin stainless steel rod to hold the specimen in the corrosion cell.

**Table 2:** Chemical composition of ASTM A706M carbon steel used in the preparation of steel specimens for electrochemical and morphological studies.

Element	C	Si	Mn	S	P	C.E.V
Content (%)	0.3	0.5	1.5	0.045	0.035	0.55

The SCPS was prepared based on the chemical composition of the concrete pore solution^26^ by adding 14.0 g of potassium hydroxide, KOH, 10.0 g of sodium hydroxide, NaOH, and 2.0 g of calcium hydroxide, Ca(OH)_2_, to 0.974 L of distilled water. Reagent grade chemicals and deionized water were utilized in the preparation of the SCPS. Also, the pH of the prepared SCPS was maintained at more than 13.4.

### Potentiodynamic polarization

**Figure 2:**
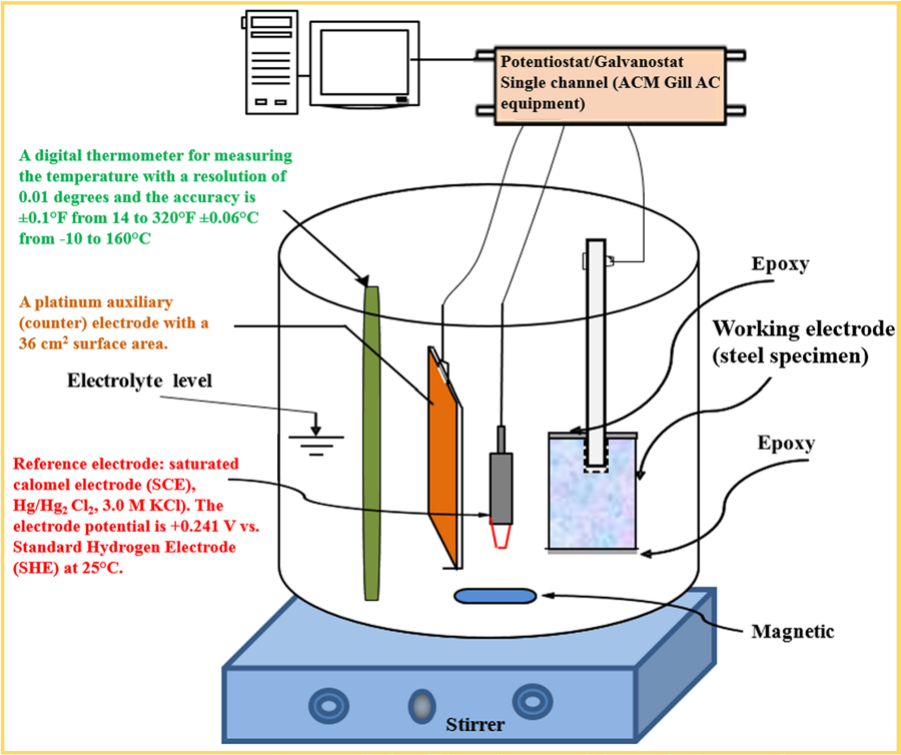
Illustration of the testing set-up used for potentiodynamic polarization study.

The carbon steel specimens were placed in a corrosion cell containing SCPS maintained at 40 °C (the selected temperature simulates the average ambient summer temperature in the hot regions of the world) and incorporating the selected concentration of chloride and sulfate ions and with or without the corrosion inhibitor. The steel specimen was immersed in SCPS, with or without the addition of the corrosion inhibitor, for 30 minutes before each experiment. Thereafter, potentiodynamic polarization (PDP) measurements were conducted using a corrosion measurement system (Fig. 2) that consisted of a computerized Potentiostat/Galvanostat (ACM equipment), magnetic stirrer, corrosion cell consisting of electrolyte, working electrode (steel specimen), counter electrode (platinum rod with a 36 cm^2^ surface area) and a reference electrode [saturated calomel electrode (SCE), Hg/Hg_2_ Cl_2_, 3.0 M KCl], and a digital thermometer for measuring the temperature of the electrolyte.

The potentiodynamic polarization (PDP) is commonly used technique to assess the mechanistic and kinetic information on corrosion of metals^29^. It measures the variation in current for the potential sweep in the cathode side as well as anode side of the potential of corrosion^29^. The PDP plots were developed by varying the electrode potential between −900 and +900 mV SCE at 15 mV/min scan rate^29,30^. At this scan rate, it takes about two hours to conduct a potentiodynamic polarization scan.

### Morphology

The steel specimens were positioned in the testing set up and potentiodynamic polarization scan was conducted. Thereafter, the surface of the samples was assessed by a Joel JSM-5800LV scanning electron microscopy.

## Results and Discussion

### Assessment of Potentiodynamic Polarization

The effect of chloride and/or sulfate ions on the corrosion of carbon steel specimens positioned in SCPS with the presence or absence of inhibitors is discussed in the next subsections.

### Effect of sulfate and chloride contamination on corrosion of carbon steel with no inhibitor

**Figure 3:**
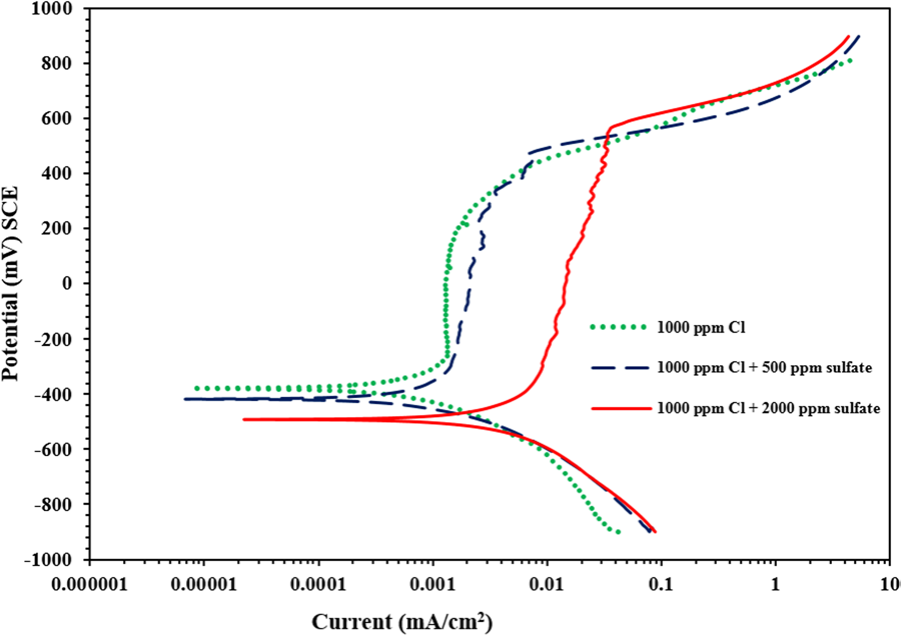
Potentiodynamic polarization plots for the carbon steel samples in SCPS; no use of any inhibitor.

Figure 3 depicts the PDP for a carbon steel specimen immersed in SCPS where no inhibitor was used and contaminated with 1000 ppm Cl and a varying sulfate concentration (0, 500 and 2000 ppm). Insignificant change in the anodic dissolution of carbon steel samples exposed to 0 and 500 ppm sulfate could be observed. However, the PDP for the carbon steel specimen in SCPS with 2000 ppm sulfate indicates an anodic behaviour compared to the specimens exposed to 0 and 500 ppm sulfate concentration. This behaviour indicates that sulfate ions influence the mechanisms of chloride-induced corrosion of carbon steel. The polarization results for the samples exposed into SCPS with no use of any inhibitor is presented in Table 3. There was an increase in corrosion current density from 1.09 to 1.55 μA/cm^2^ with the change of sulfate concentration from 0 to 2000 ppm.

**Table 3:** Potentiodynamic polarization results for carbon steel inside SCPS; no use of corrosion inhibitor (control).

Chloride + sulfate concentration (ppm)	E_corr_ (mV SCE)	Rp (kΩ.cm^2^)	I_corr_ (μA/cm^2^)	Rate of corrosion (mm/y)	Increase in corrosion rate due to sulfate, (%)
1000 + 0	−377	23.84	1.09	0.0127	—
1000 + 500	−418	23.07	1.13	0.0131	3
1000 + 2000	−496	16.85	1.55	0.0179	29

### Corrosion of carbon steel in SCPS and calcium nitrite-based proprietary inhibitor I

**Figure 4:**
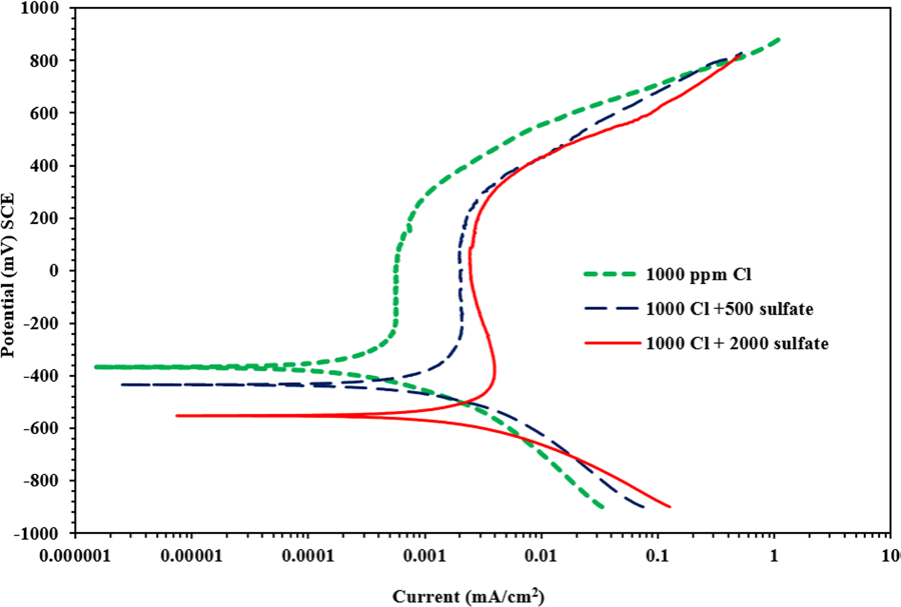
Potentiodynamic polarization plots for carbon steel in SCPS with inhibitor I.

Figure 4 presents the PDP for carbon steel samples exposed to SCPS with the incorporation of inhibitor I and contaminated with 1000 ppm Cl plus 0, 500 or 2000 ppm SO_4_. Almost similar corrosion was observed in the samples. The corrosion potential decreased from −366 to −555 mV SCE with an increase in the sulfate concentration from 0 to 2000 ppm. The decrease in the corrosion potential indicates an enhancement in the inhibition performance. The anodic dissolution of the carbon steel specimen exposed to 2000 ppm sulfate, which was more anodic in the absence of an inhibitor, became less anodic when inhibitor I was used, thereby indicating that this inhibitor was able to mitigate the corrosion of the carbon steel sample exposed to both chloride and chloride plus sulfate solutions.

The potentiodynamic data for the carbon steel sample inside SCPS with inhibitor I are listed in Table 4. There was an increment in the corrosion current density between 0.14 to 0.31 µA/cm^2^ due to the change in the sulfate level from 0 to 2000 ppm.

**Table 4:** Potentiodynamic polarization results for carbon steel immersed in SCPS with the incorporation of calcium nitrite based inhibitor I (Proprietary liquid concrete mixture; dosage of 15 L/m^3^).

Chloride + sulfate concentration (ppm)	E_corr_ (mV SCE)	Rp (kΩ.cm^2^)	I_corr_ (μA/cm^2^)	Rate of corrosion (mm/y)	Efficiency (%)
1000 + 0	−366	193.54	0.135	0.0016	88
1000 + 500	−436	154.4	0.169	0.0020	85
1000 + 2000	−554	84.3	0.31	0.0036	80

The inhibition effectiveness η_*E*_(%)of the investigated inhibitors was computed as:

1 $${{\rm{\eta }}}_{E}=(\frac{{{\rm{i}}}_{{\rm{o}}}-{\rm{i}}}{{{\rm{i}}}_{{\rm{o}}}})\times 100$$

where, i is the corrosion current density without the use of inhibitor; and i_o_ is the corrosion current density when an inhibitor was used.

### Corrosion of carbon steel exposed to SCPS with Calcium nitrite-based inhibitor II

**Figure 5:**
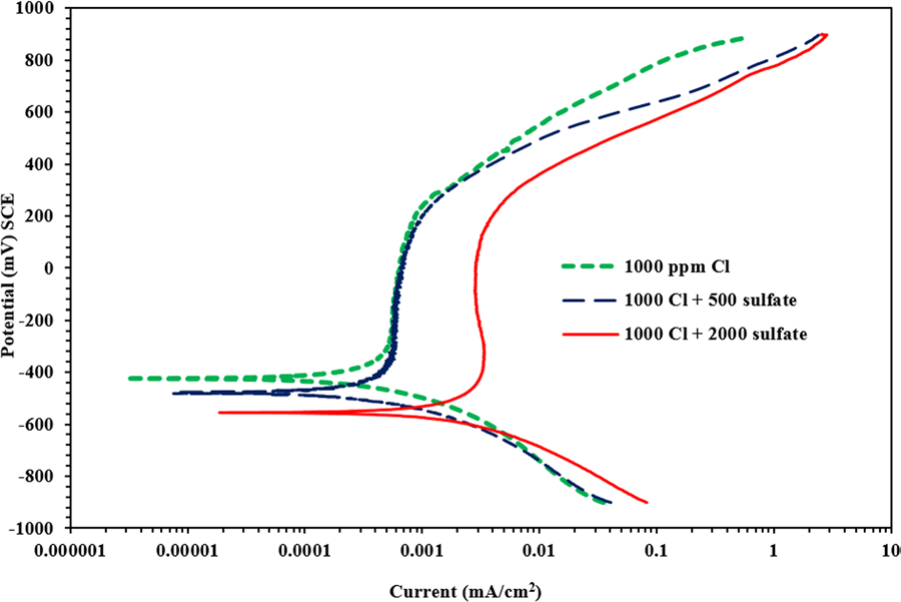
Potentiodynamic polarization plots for carbon steel in SCPS with generic corrosion calcium nitrite-based inhibitor II.

The PDP plots for carbon steel samples in SCPS with inhibitor II are presented in Fig. 5. Almost similar corrosion was shown in the samples exposed to both systems, i.e. chloride or chloride plus sulfate ions. The corrosion potential decreased from −419 to −555 mV SCE with an increase in the SO_4_ concentration from 0 to 2000 ppm. Further, the current required for the transition from the anodic to the cathodic regions was less in the specimens that were exposed to 2000 ppm SO_4_ than those exposed to 500 ppm SO_4_ (0.061 and 0.069 µA/cm^2^, respectively). A lower transition current is indicative of increased corrosion activity due to an increase in the sulfate concentration.

The PDP results for carbon steel samples exposed to SCPS with inhibitor II are listed in Table 5. There was an increment in the current density of corrosion from 0.17 to 0.44 µA/cm^2^ with increasing the sulfate from 0 to 2000 ppm. The efficiency of this inhibitor decreased between 84% to 72% with changing the concentration of sulfate between 0 and 2000 ppm.

**Table 5:** Potentiodynamic polarization data for carbon steel immersed in SCPS with generic corrosion calcium nitrite-based inhibitor II (; dosage of 15 L/m^3^).

Chloride + sulfate concentration (ppm)	E_corr_ (mV Vs SCE)	Rp (kΩ.cm^2^)	I_corr_ (μA/cm^2^)	Rate of corrosion (mm/y)	Efficiency (%)
1000 + 0	−419	155.31	0.1680	0.0019	85
1000 + 500	−474	83.33	0.3130	0.0036	72
1000 + 2000	−554	59.94	0.4352	0.0050	72

### Corrosion of carbon steel exposed to SCPS with Proprietary amine carboxylate-based inhibitor III

**Figure 6:**
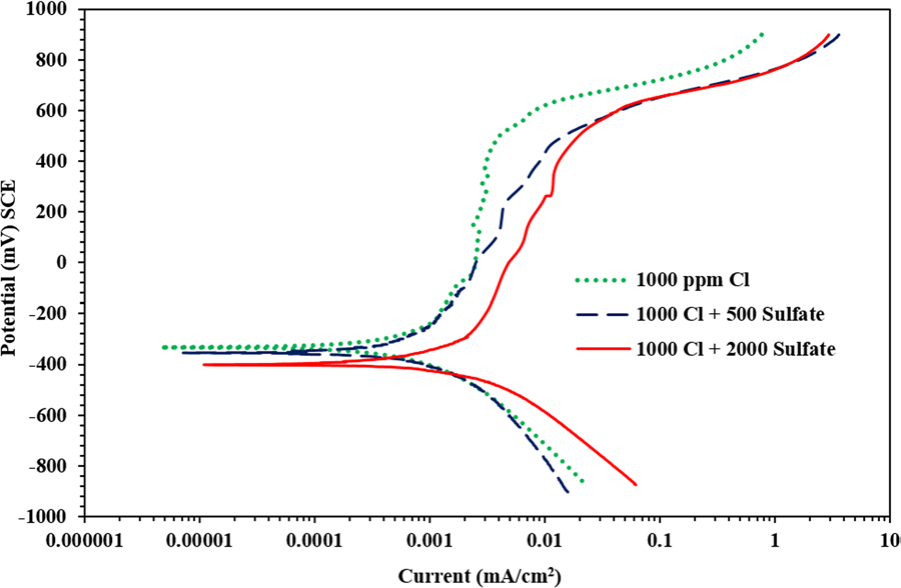
Potentiodynamic polarization plots for carbon steel in SCPS with the proprietary liquid concrete mixture with amine carboxylate-based inhibitor III.

The PDP plots for carbon steel samples immersed in SCPS with the incorporation of inhibitor III (Proprietary Amine Carboxylate-based) are shown in Fig. 6. Again, almost the same corrosion was observed in the samples. The corrosion potential decreased from −334 to −402 mV SCE as the sulfate concentration increased from 0 to 2000 ppm. Further, the current density required for the transition from the cathodic to the anodic region varied from 0.079 to 0.043 μA/cm^2^ due to changing the SO_4_ from 500 to 2000 ppm.

The polarization measurements for this set of specimens are listed in Table 6. There was an increase in the current density between 0.17 to 0.47 µA/cm^2^ with the increase the amount of sulfate from 0 to 2000 ppm. The effectiveness of inhibitor III reduced from 85% to 70% as the sulfate concentration increased from 0 to 2000 ppm. It is apparent that the efficiency of this inhibitor decreases due to an increase in the sulfate concentration; although it should be noted that this inhibitor is effective in reducing the current density of corrosion.

**Table 6:** Potentiodynamic polarization results for carbon steel immersed in SCPS with he incorporation of proprietary liquid concrete mixture amine carboxylate-based inhibitor III (dosage: 0.6 L/m^3^).

Chloride + sulfate concentration (ppm)	E_corr_ (mV Vs SCE)	Rp (kΩ.cm^2^)	I_corr_ (μA/cm^2^)	Rate of corrosion (mm/y)	Efficiency (%)
1000 + 0	−336	155.27	0.1680	0.0019	85
1000 + 500	−360	93.18	0.2800	0.0032	75
1000 + 2000	−402	55.26	0.4721	0.0055	70

### Corrosion of carbon steel exposed to SCPS and proprietary modified amino alcohol-based inhibitor IV

**Figure 7:**
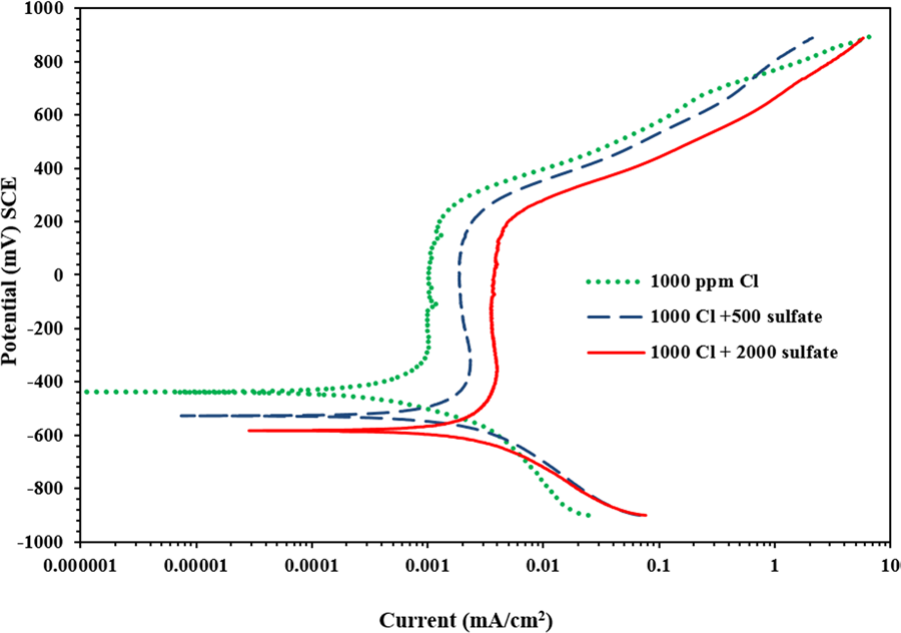
Potentiodynamic polarization plots for carbon steel in SCPS with amino alcohol base inhibitor IV (Proprietary liquid concrete mixture).

Figure 7 depicts the PDPs for carbon steel samples exposed to SCPS with the incorporation of inhibitor IV and contaminated with 1000 ppm Cl and 0, 500 and 2000 ppm SO_4_. As shown in Table 7, there was an increment in the corrosion current density from 0.25 to 1.12 μA/cm^2^ with the change in sulfate concentration between 0 to 2000 ppm. Further, the inhibitor efficiency decreased sharply from 78% to 28% as the sulfate concentration changed from 0 to 2000 ppm. Despite its superior performance in the chloride environment, this inhibitor does not perform well in the chloride plus sulfate environment.

**Table 7:** Potentiodynamic polarization data for carbon steel immersed in SCPS with incorporation of modified amino alcohol-based inhibitor IV (Proprietary liquid concrete mixture; dosage 15 L/m^3^).

Chloride + sulfate concentration (ppm)	E_corr_ (mV SCE)	Rp (kΩ.cm^2^)	I_corr_ (μA/cm^2^)	Rate of corrosion (mm/y)	Efficiency (%)
1000 + 0	−376	106.25	0.246	0.0029	78
1000 + 500	−482	36.87	0.707	0.0082	38
1000 + 2000	−523	23.34	1.118	0.0130	28

### Corrosion of carbon steel exposed to SCPS and inhibitor V **(**Proprietary calcium nitrite-based inhibitor)

**Figure 8:**
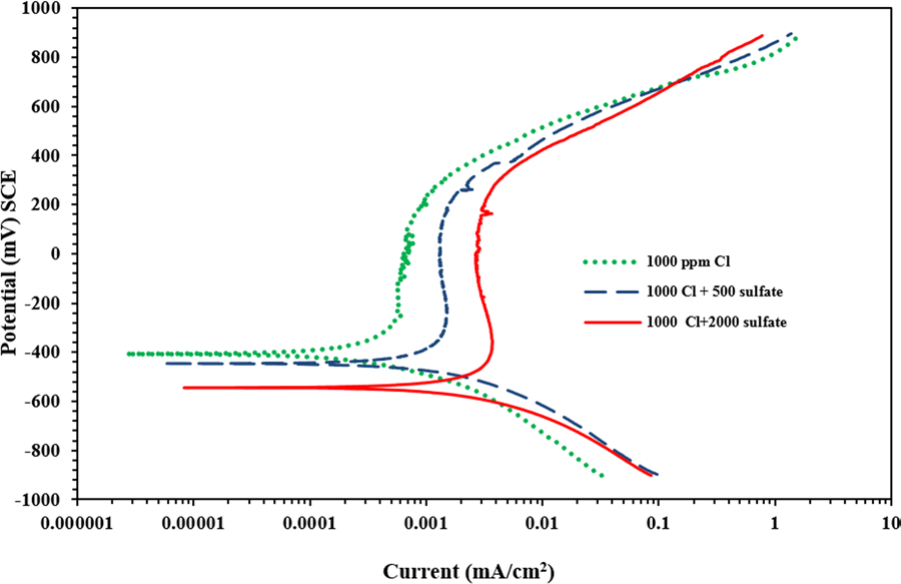
Potentiodynamic polarization plots for carbon steel in SCPS with calcium nitrite-based inhibitor V (Proprietary liquid concrete mixture).

Figure 8 depicts the PDP plots for carbon steel samples immersed in SCPS with inhibitor V and 1000 Cl and 0, 500 or 2000 ppm SO_4_. The corrosion potential for carbon steel samples in SCPS with 0, 500 and 2000 ppm SO_4_ was −405, −447 and −541 mV SCE, respectively. Further, uniform corrosion was observed and the polarization data are listed in Table 8. There was a change in I_corr_ from 0.16 to 0.33 μA/cm^2^ with changing the level of SO_4_ from 0 to 2000 ppm. The efficiency of this inhibitor decreased marginally from 86% to 79% due to an increase in the sulfate concentration.

**Table 8:** Potentiodynamic polarization results for carbon steel immersed in SCPS with incorporation of calcium nitrite based inhibitor V (Proprietary liquid concrete mixture; Dosage of 15 L/m^3^).

Chloride + sulfate concentration (ppm)	E_corr_ (mV vs SCE)	Rp (kΩ.cm^2^)	I_corr_ (μA/cm^2^)	Rate of corrosion (mm/y)	Efficiency (%)
1000 + 0	−404	166.15	0.1570	0.0018	86
1000 + 500	−446	118.75	0.2197	0.0025	81
1000 + 2000	−540	79.31	0.3290	0.0038	79

It is apparent from the PDP data that all the investigated inhibitors were efficient in reducing the I_corr_ on the carbon steel samples in SCPS with 1000 ppm Cl^−^ by around four to eight times. However, when the sulfate ions were added to the SCPS, inhibitors I, II, III, and V were effective in decreasing the I_corr_ by 3.3 to 6.7 times (depending on the sulfate concentration). In the case of inhibitor IV, the I_corr_ decreased by only 1.4 to 1.6 times (again depending on the sulfate concentration). This indicates that this inhibitor was marginally effective in mitigating corrosion in the combined presence of sulfate and chloride ions.

Generally, an increase in the sulfate concentration from 0 to 2000 ppm significantly increased the anodic dissolution of carbon steel (see Fig. 3 through 8); indicating that the sulfate or chloride tends to significantly accelerate the rate of corrosion. There was an increment in the current density of the steel in SCPS from 1.3 to 3.5 times with increment in the sulfate level from 0 to 2000 ppm (see Tables 4 through 8). However, the incorporation of the selected inhibitors (organic and inorganic) decreased the corrosion rate of carbon steel.

**Figure 9:**
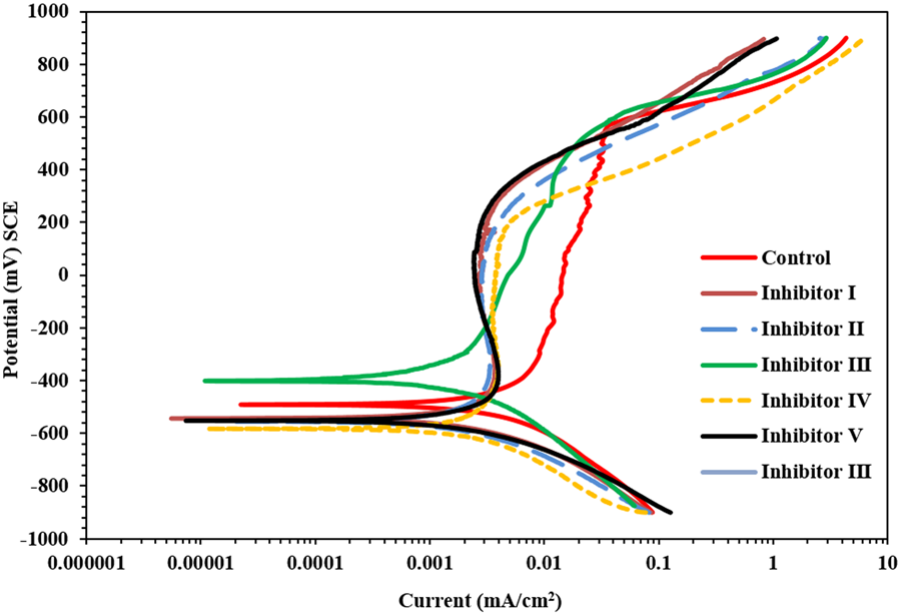
Potentiodynamic polarization plots for carbon steel in SCPS contaminated with 2000 ppm SO_4_ and 1000 ppm Cl.

Figure 9 depicts the PDPs for carbon steel samples exposed to SCPS with and without corrosion inhibitors and contaminated with 1000 ppm Cl and 2000 ppm SO_4_. Same corrosion was observed in the tested samples. There was a significant and uniform rise in the anodic dissolution for the control specimens (carbon steel samples exposed to SCPS without corrosion inhibitor and with 1000 ppm Cl and 2000 ppm SO_4_). However, the PDP for the carbon steel specimen exposed to SCPS, incorporating corrosion inhibitors and contaminated with 1000 ppm Cl and 2000 ppm SO_4,_ was less anodic than on the control specimens. Further, the current required for the transition from the anodic to the cathodic regions was less in the control sample than on the samples that were exposed to SCPS incorporating corrosion inhibitors. Also, a lower transition current is indicative of increased corrosion activity.

The results of the PDP measurements, as well as the comparison plots depicted in Fig. 9, revealed that the effectiveness of corrosion inhibition of the studied inhibitors was in the order: inhibitor - I > inhibitor - V > inhibitor - III > inhibitor - II > inhibitor - IV.

### Morphology of carbon steel specimens immersed in SPCS with chloride and sulfate

**Figure 10:**
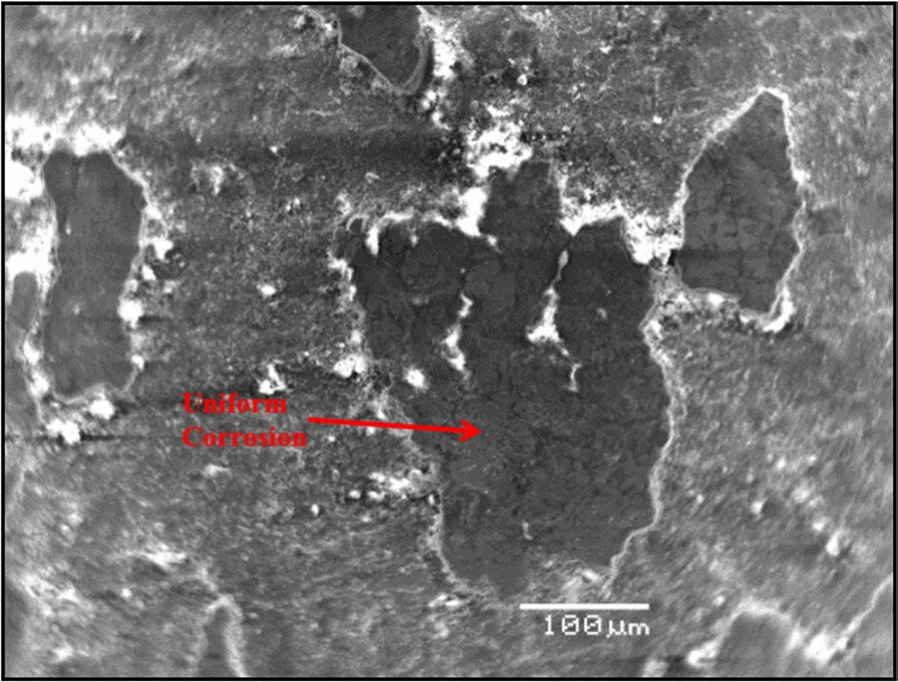
SEM of carbon steel in SCPS contaminating with 1000 ppm Cl plus 2000 ppm SO_4_.

**Figure 11:**
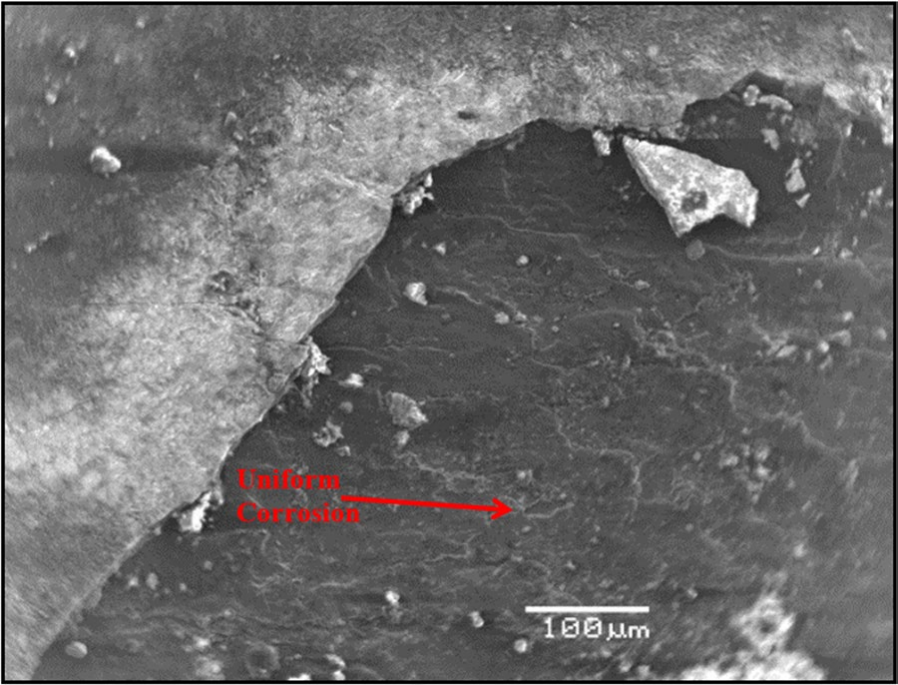
SEM of carbon steel in SCPS with incorporation of calcium nitrite based inhibitor I (liquid concrete mixture) and contaminated with 1000 ppm Cl plus 2000 ppm SO_4_.

**Figure 12:**
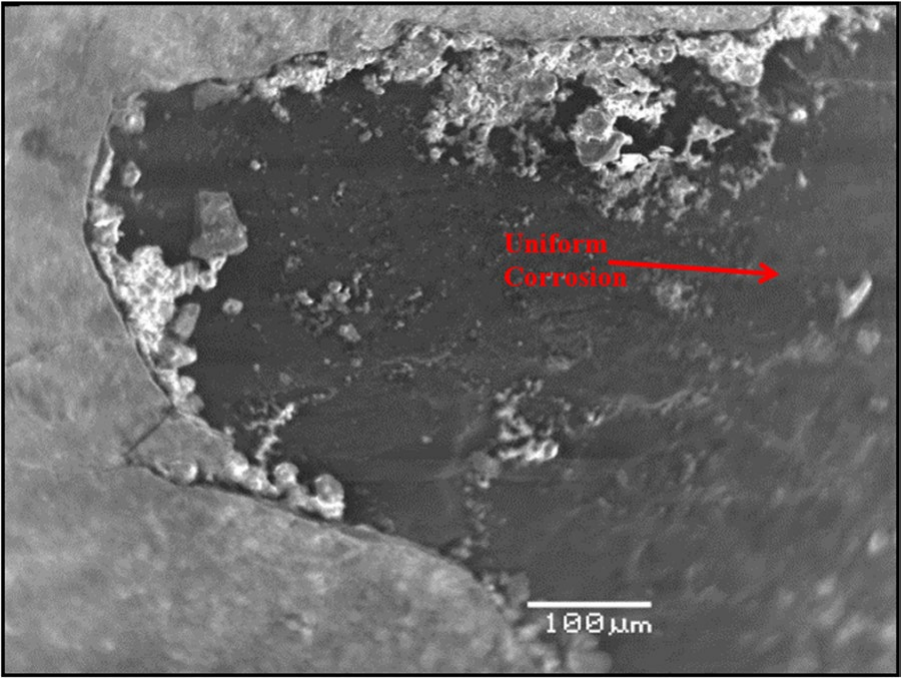
SEM of carbon steel in SCPS with the incorporation of calcium nitrite based inhibitor II (generic corrosion inhibitor) and contaminating with 1000 ppm Cl plus 2000 ppm SO_4_.

**Figure 13:**
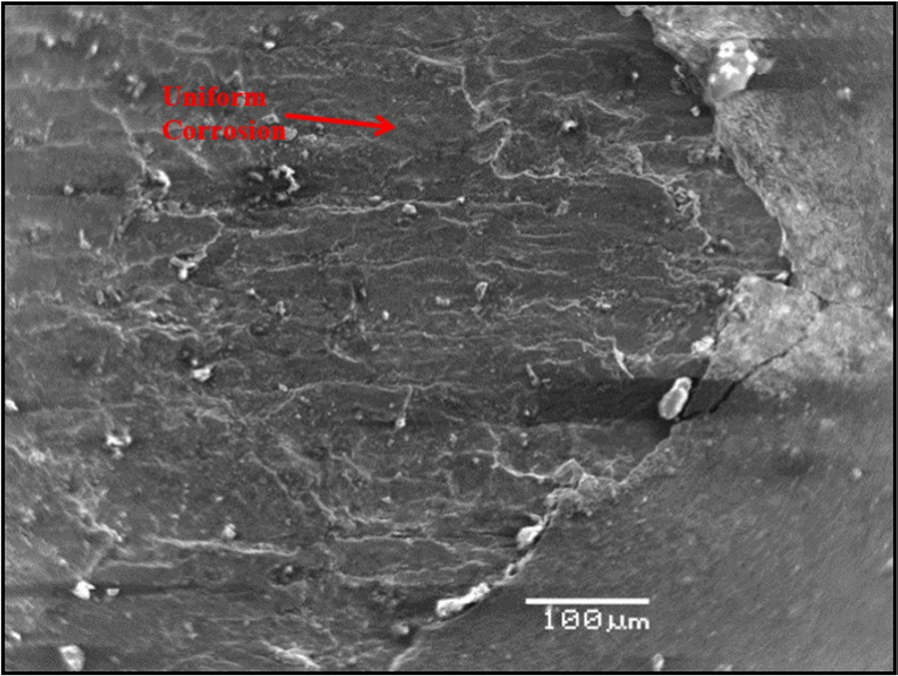
SEM of carbon steel in SCPS with the incorporation of amine carboxylate based inhibitor III (liquid concrete mixture) and contaminating with 1000 ppm Cl plus 2000 ppm SO_4_.

**Figure 14:**
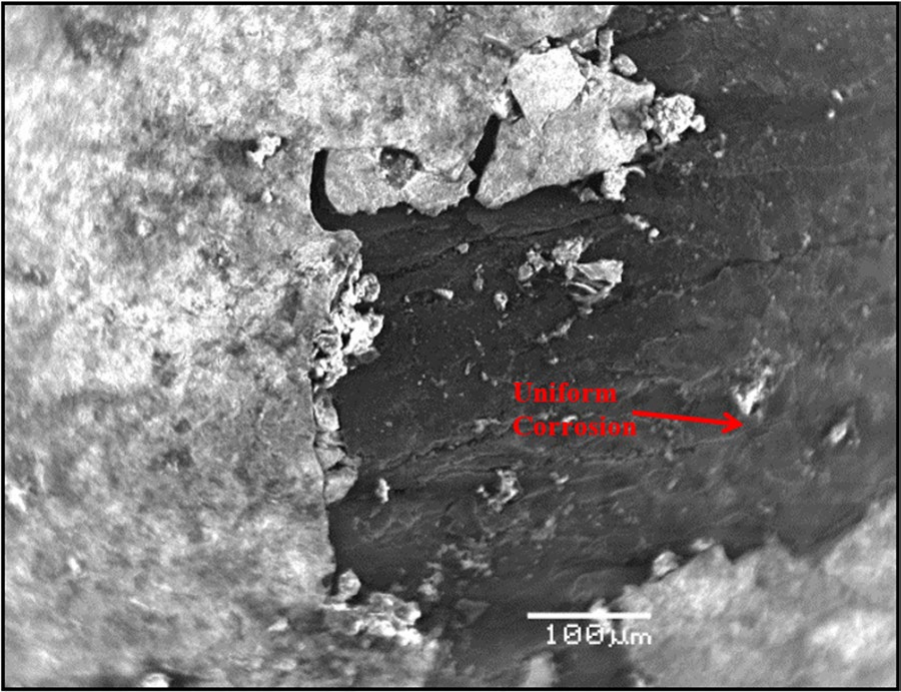
SEM of carbon steel in SCPS with the incorporation of amino alcohol-based inhibitor IV (liquid concrete mixture) and contaminating with 1000 ppm Cl plus 2000 ppm SO_4_.

**Figure 15:**
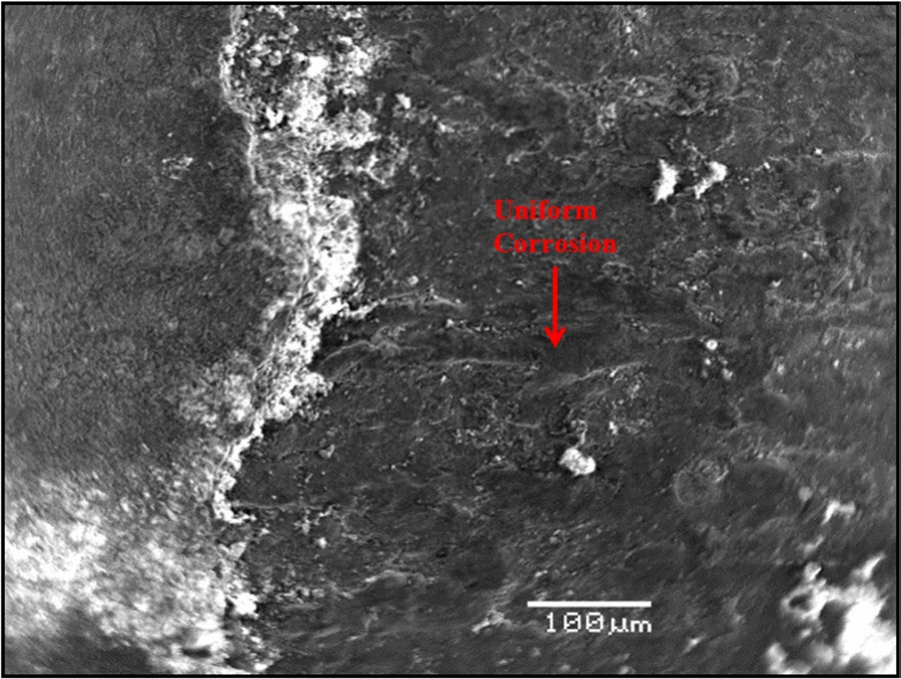
SEM of carbon steel in SCPS with the incorporation of calcium nitrite-based inhibitor V (liquid concrete mixture) and contaminating with 1000 ppm Cl plus 2000 ppm SO_4_.

Figure 10 shows the morphology of the carbon steel specimen immersed in SCPS with no corrosion inhibitor and with 1000 ppm Cl plus 2000 ppm sulfate. Uniform corrosion was observed of the specimen. In the presence of inhibitors, marginal localized corrosion and a good protective film were noted on the surface of the specimens (Figs 11–15). The morphology of the steel specimens indicates that the investigated inhibitors were effective in reducing the corrosion of carbon steel. The surface of the carbon steel samples in SCPS with the incorporation of the selected inhibitors was more adherent and thinner than that on the control specimen, i.e. without an inhibitor. Further, the corrosion product on the control specimen, i.e., without an inhibitor, was loose and less adherent.

### Mechanisms of inhibition

**Figure 16:**
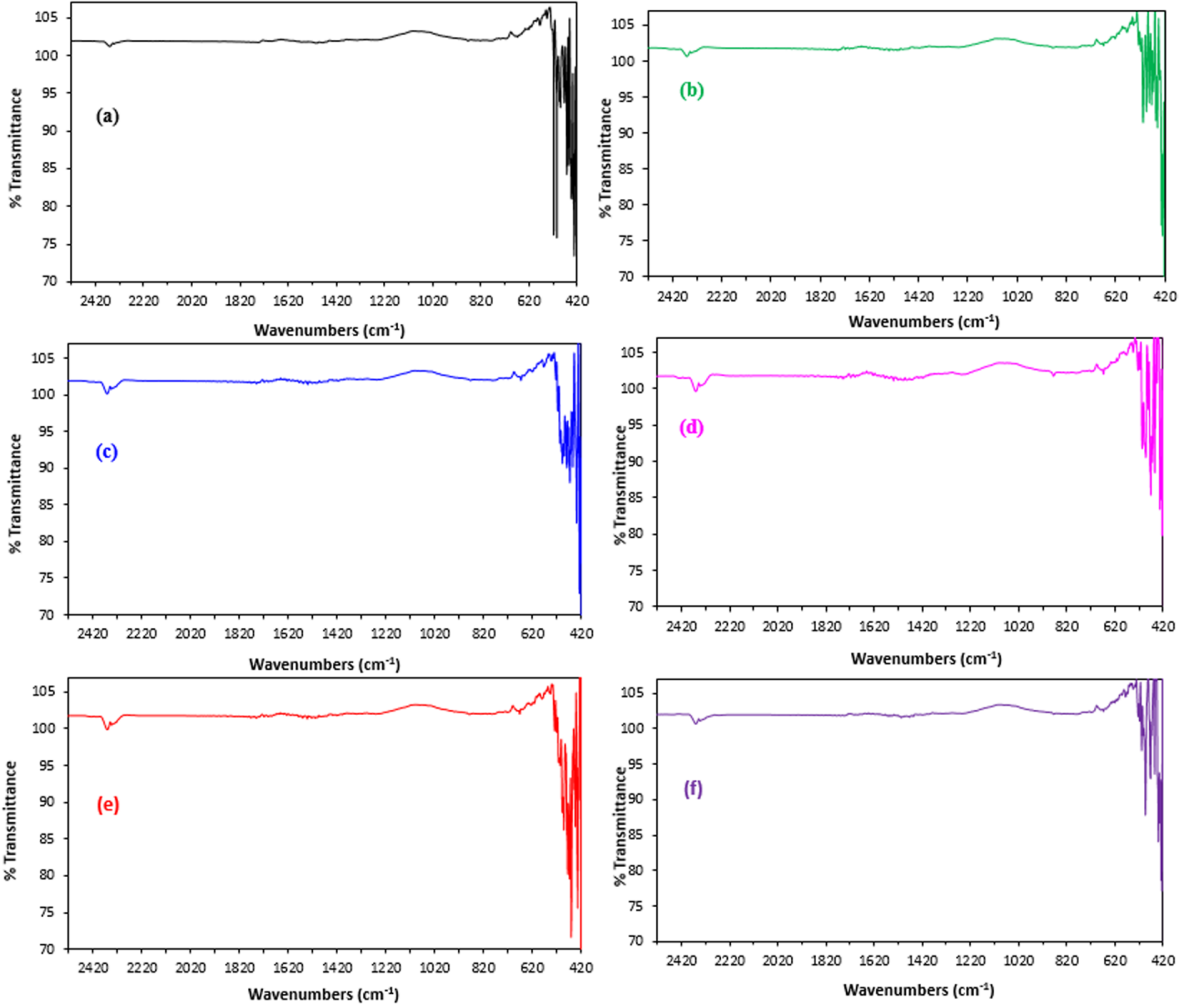
FTIR spectra of carbon steel in SCPS and contamination with 1000 ppm Cl plus 2000 ppm SO_4_ (**a**) without inhibitor; and incorporating inhibitor (**b**) I, (**c**) II, (**d**) III, (**e**) IV, (**f**) V.

Thermo Scientific Nicolet iS10 Smart iTR Fourier transform infrared spectroscopy was utilized to obtain IR spectra (Fig. 16) of the specimens to observe the iron oxide formed on the corroded steel specimens. Formation of iron oxide was observed on the metal surface on the samples exposed to SCPS with no inhibitor and with inhibitor II and inhibitor IV. The high intensity of the peaks assigned to Fe‒O bond, between 450 to 650 cm^−1^ in the spectra of the control specimen and with inhibitor IV, indicates the increased formation of iron oxides compared to the samples exposed to SCPS incorporating inhibitors I or V. The FTIR results (Fig. 16) support the PDP and SEM observations.

**Figure 17:**
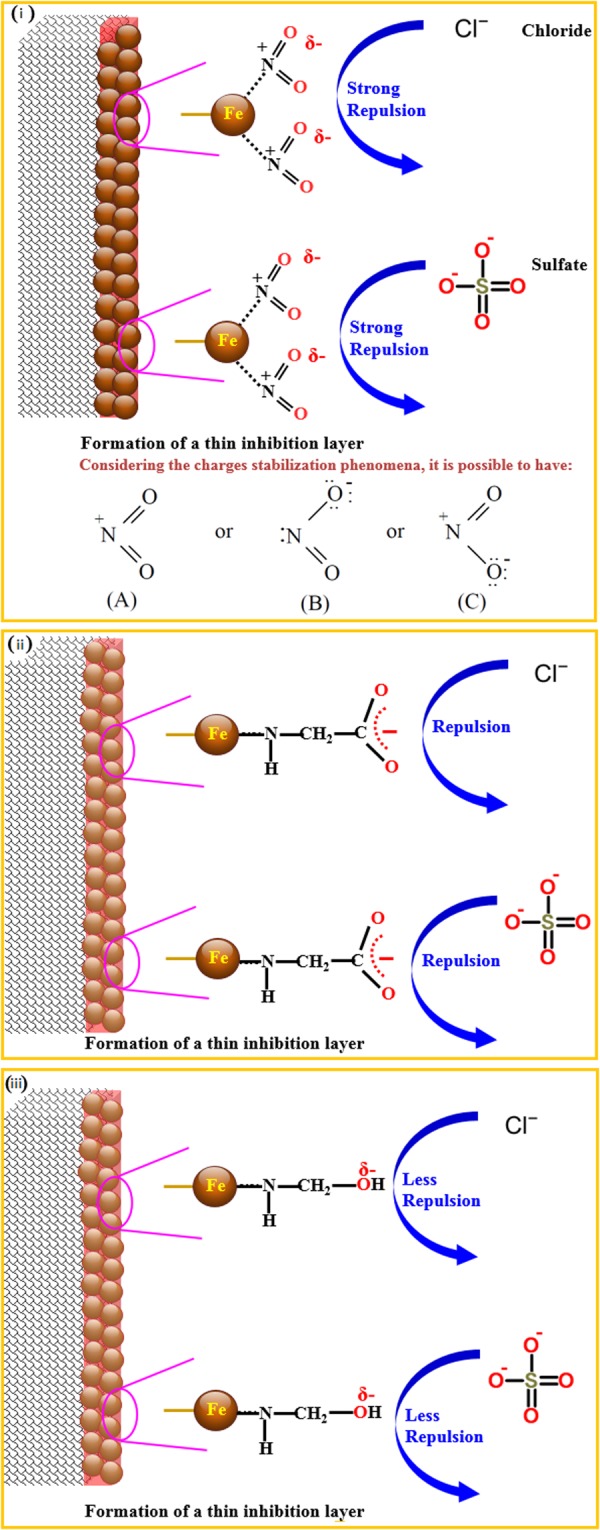
Proposed mechanisms for the inhibition of corrosion of carbon steel by the investigated: (i) nitrite-, (ii) amine carboxylate-; and (iii) modified amino alcohol-based inhibitors.

The data on inhibition efficiency indicate that the nitrite-based inhibitors performed better than the inhibitor based on amine carboxylate which performed better than the amino alcohol based inhibitor. The observed trend can be chemically explained as illustrated in Fig. 17. The nitrite-based inhibitors form an inhibition layer surrounding carbon steel^28^. As illustrated in Fig. 17(i), nitrogen (N) may interact with the carbon steel while the oxygen (O) forms a negative layer on the outer surface which at the end leads to form a protective film. To have homogeneous monolayer protective film formation on the steel surface acting as a barrier coming from mass and charge transfer, the adsorption relies on the interaction between particle and electrode which occurs from the specimen (B) by the action of the unshared electrons pair of the nitrogen atom. Another particle-electrode interaction should be considered coming from the strong negative charge of oxygen [on the specimens (B) and (C)] as the more electronegative constituent of the inorganic compound (calcium nitrite). Thus, the negative surface can act in the repulsion of the chloride and sulfate ions because of their negative charge.

On the other hand, it can be hypothesized that the amine carboxylate based inhibitors, Fig. 17(ii), could form a film on the steel surface by interacting nitrogen with iron atoms while an outer negative surface can be formed by carboxylates. Thus, building up a repulsion force between both the chloride in the environment or media and the carboxylate. While this force is strong at low concentrations, the repulsion force became weak at high amounts of chloride and sulfate. Thus, the decrease in efficiency of the film can be attributed to the possible penetration of chloride or sulfate to the steel.

On the other side, the lower efficiency of the amino alcohol based inhibitor can be explained by having only one hydroxyl group which forms a lower negative charge on the carbon steel, thus, forming a weak film on the steel, as schematically presented in Fig. 17(iii), compared with the nitrite- and carboxylate-based inhibitors.

## Conclusions

The effectiveness of all the investigated corrosion inhibitors decreased marginally with an increase in the sulfate concentration from 0 to 2000 ppm. While the effectiveness of amino alcohol-based inhibitor IV, in chloride plus sulfate, decreased sharply with changing the concentration of sulfate from 0 to 2000 ppm. Thus, this inhibitor IV does not perform well in the chloride plus sulfate environment. It can be concluded that the inhibiting effect of the nitrite-based inhibitors, under chloride and sulfate environments, was superior to that of the amine carboxylate- and amino alcohol-based inhibitors, which can be ascribed to the varying chemical structure of the functional groups. The nitrite-based inhibitors form a protective layer surrounding the carbon steel which promotes the repulsion of the chloride and sulfate. This film is stronger than the film formed in the case of organic inhibitors based on amine carboxylate and amino alcohol due to the higher negative charge. A loose and non-adherent corrosion product was observed on the samples in SCPS only with no inhibitor, while a thin layer of a well adherent corrosion product was noted on the steel specimens which incorporated the investigated inhibitors. Thus, the incorporation of the inhibitors not only decreases the rate of corrosion, but it also produces a more adherent corrosion product that is beneficial in inhibiting further corrosion.
